# Occipital nerve stimulation for cluster headache: lessons to learn from the ‘voltage tuners’

**DOI:** 10.1186/s10194-024-01839-7

**Published:** 2024-08-23

**Authors:** Linda Kollenburg, H. Arnts, M. Heitkamp, S. Geerts, C. Robinson, M. Dominguez, W. Mulleners, E. Kurt

**Affiliations:** 1 Department of Neurosurgery, Radboud University Medical Center, Geert Grooteplein Zuid 10, Nijmegen, 6525 GA Netherlands; 2 Department of Neurology, Canisius Wilhelmina Hospital (CWZ), Nijmegen, Netherlands; 3 Department of Anesthesiology, Perioperative, and Pain Medicine, Harvard Medical School, Brigham and Women’s Hospital, Boston, MA USA; 4 Department of Neurology, New York Presbyterian Hospital, Weill Cornell Medical College, New York, NY USA; 5 Department of Neurology, Radboud University Medical Center, Nijmegen, The Netherlands

**Keywords:** Occipital nerve stimulation, Cluster headache, Voltage, Amplitude, Voltage tuner, Voltage tuning

## Abstract

**Background:**

Cluster headache (CH) is a significant health concern due to its major socioeconomic consequences and most patients being refractory to conventional strategies. For treatment resistant CH, occipital nerve stimulation (ONS) is considered an effective treatment option. Whereas most patients do not adjust the amplitude of the ONS system, a subset changes the amplitude on a regular basis using their remote control, and are therefore referred to as ‘voltage tuners’. Anxiety and self-control are thought to be central themes to this behavior. Research on this voltage tuning behavior could provide new insights in the use of ONS as acute attack treatment. To date, voltage tuning has not been assessed for CH. Hence this is a unique study aiming to investigate the occurrence and efficacy of voltage tuning in patients with CH and ONS.

**Methods:**

For this analysis, patients with CH who received ONS from 2020–2024, at our university medical center, were included. All patients underwent bilateral ONS implantation. Data on attack frequency, intensity and duration were collected retrospectively. Outcomes on the response, frequency, moment during the day, duration, rationale, sensation, average increase in amplitude, and efficacy of voltage tuning were collected with prospective interviews.

**Results:**

Thirty-three patients (M = 20) (42 ± 12.7 years) were included in the current analysis. At 1y follow-up, an overall response rate of 70% (23/33) was found for ONS. In total, 48% (18/33) of patients were defined as voltage tuners. Voltage tuning was performed with an average increase in amplitude of 92 (20–360)%, a frequency of 1–20 times/month and duration of 20 minutes-48 hours. Sensations of voltage tuning were described as “tingling” and/or “pinching”. The rationale for voltage tuning in patients varied from prevention and ceasing to lowering the intensity and enhance control of CH attack.

**Conclusions:**

Outcomes show that voltage tuning may cease and/or terminate CH attacks and therefore raise interests in the use of ONS as acute attack treatment for patients with resistant CH treated with ONS. Future research on the occurrence and potential of voltage tuning will provide valuable insights for achieving optimal efficacy of ONS and quality of life in patients with CH.

## Graphical abstract

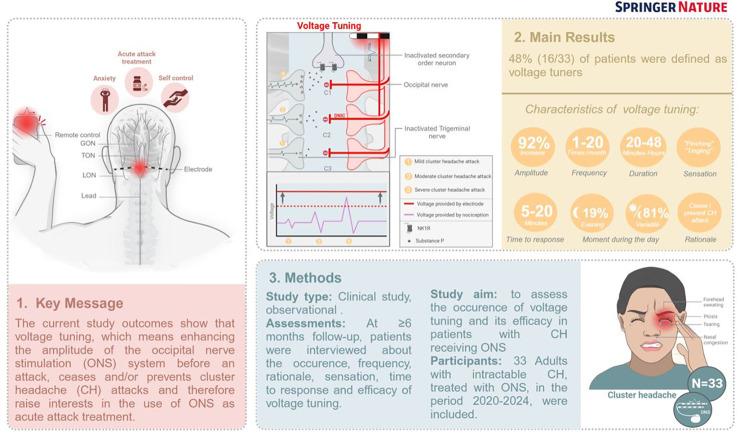

## Introduction

Cluster headache (CH) is considered a global health concern, affecting approximately 0.1% of the general population [1, 2]. CH is characterized by side-locked, excruciating attacks in the distribution of the trigeminal nerve accompanied by ipsilateral cranial autonomic symptoms, such as lacrimation, nasal congestion, and a sense of restlessness and agitation [3]. Although the exact mechanism underlying CH is poorly understood, it is thought that the hypothalamus, trigeminovascular system and autonomic system are involved [4]. It has also been suggested that disturbances in the occipital nerve may play an additional role [5]. The complex pathophysiology of CH is one of the reasons that drug resistance is present in a subset of patients [6]. Since the 1970s, occipital nerve stimulation (ONS) has become a possible treatment option in cases of drug-resistant refractory chronic CH [7, 8]. Studies have demonstrated that ONS is an effective treatment option for medically refractory chronic headache [2, 9–11]. ONS requires the implantation of electrodes, leads and an implantable pulse generator (IPG), allowing for electrical stimulation of the occipital nerve. Following the procedure, a trained physician or nurse adjusts the parameter setting of the ONS system to find the best setup. Patients have a handheld remote control, allowing them to adjust the amplitude manually. With regard to use of the remote control, there are two groups of patients: one who does not adjust the amplitude and another who does, with the latter being termed ‘voltage tuners’. Patients classified as voltage tuners tend to rapidly increase the amplitude of the occipital nerve stimulator whenever they feel a CH attack might be coming. Despite the rationale to this voltage tuning behavior before CH attacks being unknown, it is suggested that prevention, anxiety, and control﻿ of the CH attacks are central themes to this behavior.

Research on voltage tuning can provide valuable insights as to how clinical outcomes of ONS can be optimized, as it allows for the investigation of ONS as an acute attack treatment. Voltage tuning may not only contribute to quality of life by ceasing or preventing CH attacks from occuring, but also by diminishing the use of alternative acute attack treatments like sumatriptan and/or oxygen which are considered invasive and are associated with various adverse events [12, 13]. This is the first observation in literature to describe this subset of individuals, hence why it can be considered a very unique and innovative topic to study. Therefore, this study aims to assess the occurrence of voltage tuning and its efficacy in patients with CH receiving ONS.

## Methods

### Study population

In this study, records from patients with medically refractory CH, treated with ONS at Radboud University Medical Center and Canisius Wilhelmina Hospital (CWZ), in the period 2020–2024 were reviewed. Patients with a minimal follow-up period of 6 months were included. The remaining inclusion and exclusion criteria were in accordance to the national guidelines for neuromodulation for intractable CH [10]. All patients had been implanted following the surgical technique as described in our previous research [2]. This study was performed according to the Dutch law and Good Clinical Practice guidelines. The Medical Review Ethics Committee region Arnhem-Nijmegen concluded that this study was not subject to the Medical Research Involving Human Subjects Act (CMO Oost-Nederland; file number: 2024–17305). All patients gave consent for using their data for the current manuscript.

### Study design and outcomes

The aims of this cohort study are to (1) assess the prevalence of voltage tuning in patients with CH undergoing ONS, (2) investigate the efficacy, amplitude adjustments, rationale, and sensations related to voltage tuning and (3) assess the efficacy of ONS. As part of the standard procedure in our hospital, patients are asked to fill a headache diary preoperatively. Preoperative baseline measurements on attack frequency, intensity, and duration were collected retrospectively from our patient database and compared to prospective follow-up data collected after ≥ 6 months follow-up. Patients were also contacted prospectively and interviewed about their attack frequency, intensity, and duration.

To assess the efficacy of ONS, patients were divided into four groups: (1) 0% reduction in attack frequency, intensity and/or duration, (2) 1–49% reduction in attack frequency, intensity, and/or duration, (3) 50–99% reduction in attack frequency, intensity, and/or duration, (4) 100% reduction in attack frequency, intensity and/or duration. Intensity of CH attack was evaluated using the numeric rating scale (NRS). Responders are defined as those having ≥ 50% reduction in attack frequency, intensity and/or duration. Patients were also asked about the occurrence of voltage tuning. Voltage tuners are defined as patients who adjust the amplitude of their IPG during their CH attacks, using their handheld remote control. If patients were classified as voltage tuners, additional questions were asked: (1) time to response, (2) frequency, (3) moment during the day, (4) duration, (5) rationale, (6) sensation and (7) efficacy of voltage tuning.

### Statistical analysis

Data on the overall proportion of responders and voltage tuners were counted manually. In case a range was reported for the frequencies and/or durations, the mean value was calculated and included in the analyses. For patients suffering bilateral attacks and performing bilateral voltage tuning, the average of both sites was calculated and used for analysis. In patients suffering unilateral attacks, only data from voltage tuning on the attack site was included.

## Results

Thirty-three patients (M = 20)(42 ± 12.7 years) were included in the current analysis. The average follow-up period was 23 ± 12.2 months. In total, 94% (31/33) of patients experienced unilateral attacks. Patients implanted before December 2022, received the Prime Advanced IPG (52%, 17/33)(Medtronic Inc., Minneapolis, MN, USA), whereas those operated afterwards, received the Vanta IPG (48%, 16/33)(Medtronic Inc., Minneapolis, MN, USA). The overall preoperative attack frequency was 7 ± 4 attacks/day with a duration of 100 ± 60 min/attack and NRS of 9 ± 1 during the attack.

Regarding the overall efficacy of ONS, 70% (23/33) of patients were defined as responders. Out of all patients, 48% (16/33) were classified as voltage tuners. In groups 1–4, 33% (1/3), 71% (5/7), 53% (9/17) and 17% (1/6) of subjects were voltage tuners respectively (Table 1). During CH attacks, subjects increased their amplitude with 92 (20–360)%, a frequency of 1–20 times/month and duration of 20 –48 h. The average increase in amplitude was 1.84 V or 0.5 mA, and, in most patients, responses to voltage tuning became apparent after 5–20 min (Table 1). Whereas some performed voltage tuning most predominantly during the evening or night (19%, 3/16), others reported variable moments (81%, 13/16) instead, meaning that the moment of voltage tuning is different each time (Table 1).

**Table 1:** Characteristics of voltage tuning in patients with CH

Patient		Responder to the ONS therapy	Age (y)	Gender	Time to response	Duration	Frequency	Moment of day	Increase in amplitude	Follow up period
		**Group 1 (0% improvement)**								
1			53	M	N/A	N/A	N/A	Variable	N/A	38 m
		**Group 2 (1–49% improvement)**								
2			48	V	< 15 m	60 m	3 times/d	Evening, night	43% (1.4 mA to 2.0 mA)	17 m
3			55	M	< 5 m	60 m	2 times/m	Variable	20% (5 V to 6 V)	27 m
4			31	V	N/A	N/A	N/A	Variable	25% (2.4 to 3.0 V)	35 m
5			40	M	< 60 m	≥ 24 h	1 time/m	Entire day	360% (1.0 V to 4.6 V)	37 m
6			46	M	N/A	N/A	N/A	Variable	N/A	6 m
		**Group 3 (50–99% improvement)**								
7^1^			45	V	< 5 m	30 m	3 times/m	Evening, afternoon	21% (1.65 V to 2 V)	38 m
8			29	M	< 15 m	≥ 24 h	2 times/m	Variable	60% (5 V to 8 V)	42 m
9			35	V	< 20 m	150 m	2 times/m	Variable	275% (1.6 V to 6 V)	29 m
10			22	M	< 20 m	≥ 24 h	2 times/m	Variable	25% (6 V to 7.5 V)	22 m
11			55	V	< 10 m	20–90 m	2 times/w	Evening, night	194% (0.85 V to 2.5)	32 m
12			45	M	< 15 m	60 m–12 h	5 times/w	Variable	122% (1.8 V to 4 V)	14 m
13			27	V	< 45 m	120 m	N/A	Variable	28% (1.8 V to 2.3 V)	12 m
14			33	M	< 30 m	30 m	4–5 times/w	Entire day	50% (2.0 V to 3.0 V)	35 m
15			33	V	< 10 m	20 m	2 times/m	Variable	20% (4.0 mA to 4.8 mA)	6 m
		**Group 4 (100% improvement)**								
16			27	M	< 120 m	48 h	2 periods/y	Entire day	49% (2.25 V to 3.35 V)	38 m

d: day; F: female; h: hour; m: month; M: male; N/A; not available; y: year; V: volts; mA: miliampíre

^1^Patient declared that less than 30 min of voltage tuning causes recurrence of CH attack

Patients described sensations of voltage tuning as ‘‘pinching’’ and/or ‘’tingling’’ and compared it to ‘‘lowering noise with a volume button’’ or ‘’a fog in the head which suddenly disappears.’’ The rationale for voltage tuning in patients varies from prevention and ceasing to lowering the intensity and enhance control of CH attack. Others mentioned that voltage tuning decreases anxiety or that it replaces painful perceptions of the CH attack with tingling sensations. None of the patients endured biological and/or technical complications from voltage tuning. With regard to adverse events for ONS in general, infection (6%, 2/33) and site pain (3%, 1/33) were reported in the current study population. Technical complications of ONS included lead migration/breakage (10%, 3/33), which were caused by a fall on the head or pressure increasing moments like extreme coughing. In total, 18% (6/33) patients required a reoperation due to technical or biological complications of the ONS system.

## Discussion

CH is characterized by severe headache attacks and therefore places a huge burden on patients lives, especially when considering that a subset of patients with chronic CH are refractory to conventional approaches. For therapy-resistant chronic CH, ONS is a treatment option. Following implantation of the ONS system, a subset of patients, referred to as ‘voltage tuners’, adjust the amplitude of the IPG on a regular basis, using their remote control. Analyzing this phenomenon will provide further insights into the use of ONS as acute attack treatment and is therefore investigated in the current study.

Outcomes of this study further support that ONS is an effective treatment option for patients with intractable CH, as an overall response rate of 70% was found. In correspondence to the results obtained in this study, others reported a response rate of 41–90% for ONS in CH [7, 14–19]. Variability in responder definition, electrode placement, imaging, patient positioning and headache intensity at baseline is likely responsible for discrepancies in outcomes on the efficacy of ONS [2, 11]. All together, due to fluctuations in symptoms of CH over time [11], variability in assessment of efficacy and surgical technique [2], analyzing and comparing outcomes of ONS remains challenging.

This research reveals that almost half of patients with CH receiving ONS were voltage tuners, emphasizing the relevance of this phenomenon. Voltage tuners were mostly present in groups 2 and 3, which can be expected considering that those with no response to ONS preferably request for an explant, and those in group 4 already having optimal results and complete reduction of CH attacks. Patients in group 4 may also not be defined voltage tuners due to anxiety for attack recurrence. A similar phenomenon of voltage tuning is also seen in spinal cord stimulation (SCS) and vagus nerve stimulation (VNS), however, it has not been reported for other types of neuromodulation such as deep brain and motor cortex stimulation [20, 21]. With regard to VNS and epilepsy, it has been shown that epileptic seizures can be terminated with a magnet, which allows for on-demand activation of stimulation by patients during seizures [21]. Various studies on SCS even recommend that patients adjust the amplitude manually as it optimizes the efficacy and resolves discomfort [22, 23]. It is a well-known phenomenon that, in patients receiving SCS, leads can dynamically migrate away from the spinal cord when performing daily activities. As a result, stimulation is experienced as less intense, thereby decreasing benefit from stimulation in the spine and extremities [24]. To prevent the dynamic lead migration from occurring, patients increase the amplitude with their handheld remote control, when performing certain activities. Increasing the amplitude in SCS leads to more intense stimulation, therefore allowing for optimal pain management regardless of the movements performed [23]. Due to recent developments of the evoked compound action potentials (ECAP), these amplitude adjustments in SCS can now be regulated automatically with a closed loop system [25].

While voltage tuning in SCS is often linked to specific activities, patients with CH being treated with ONS, report more variable moments, not linked to specific activities. Some manually adjust the amplitude in the evening or night, whereas others report variable moments during the day (Table 1). The moment of voltage tuning is likely dependent on the onset of CH attacks, which varies among patients. Hence, the rationale of voltage adjustments in SCS likely differs from ONS, as the leads are placed in the occipital rather than spinal region, and therefore do not move as much during during activities.

**Fig. 1:**
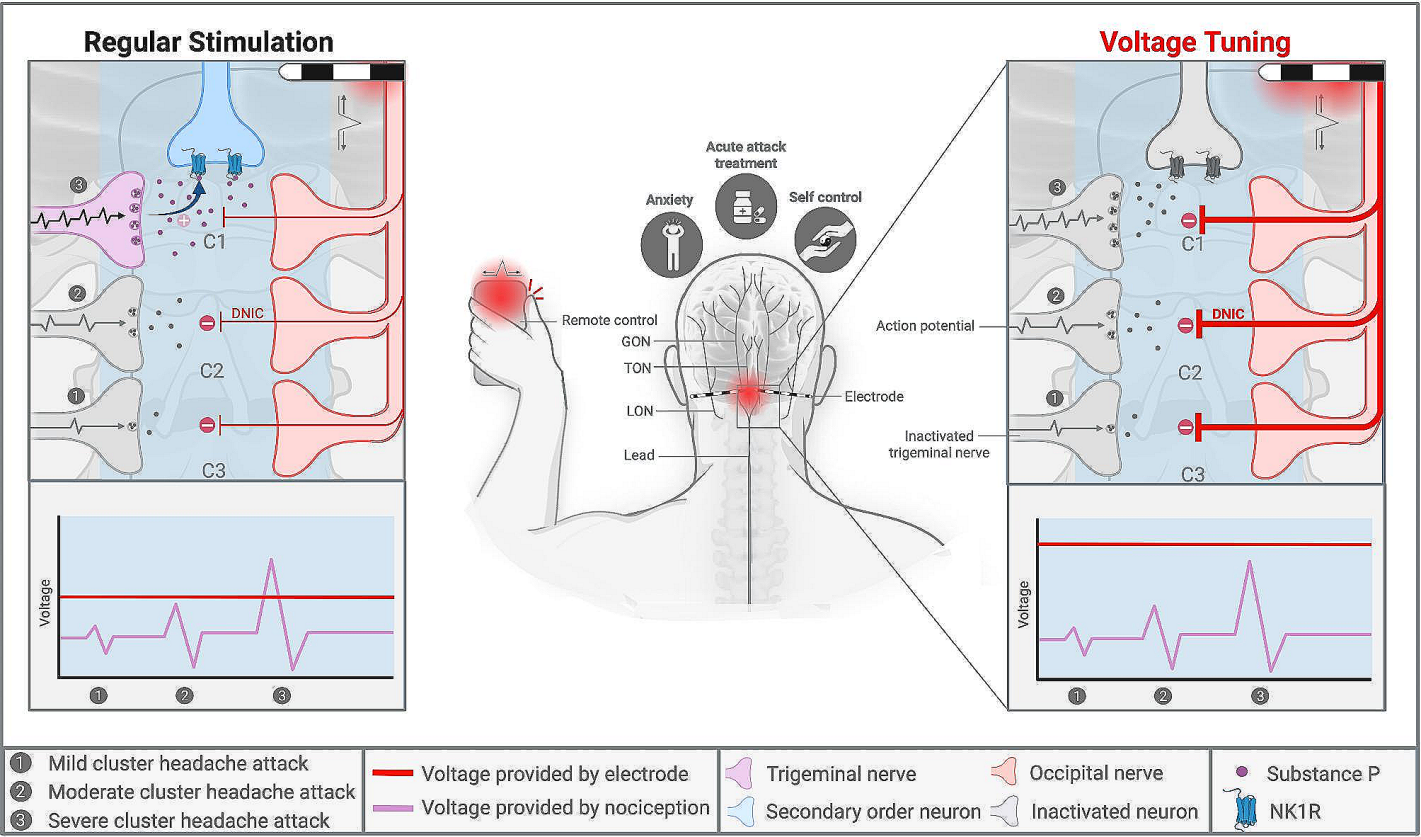
Overview of mechanism underlying occipital nerve stimulation and voltage tuning in patients with cluster headache. In ‘regular stimulation’, amplitudes are set to lower values, consequently leading to prevention of mild and moderate, but not severe cluster headache attacks. In ‘voltage tuning’, amplitudes are temporary set to higher values, leading to prevention of mild, moderate and severe cluster headache attacks. C: cervical vertebrae; DNIC: diffuse noxious inhibitory control; GON: greater occipital nerve; LON: lesser occipital nerve; NK1R: neurokinin-1 receptor; TCC: trigeminal cervical complex; TON: third occipital nerve

With regard to the mechanism of ONS, it is thought that the pain in CH attacks is attenuated by diffuse noxious inhibitory control (DNIC) in the trigeminal cervical complex (TCC), leading to decreased activation of the trigeminal vascular pathway via the secondary order neuron, in response to nociceptive stimuli (Fig. 1) [2, 26]. DNIC refers to an altered response to painful sensations due to administration of electrical stimulation [26]. Though not previously investigated, a plausible explanation for the beneficial effects seen in voltage tuning, could be enhancements in the overall mechanism of ONS. Voltage tuning may increase DNIC and therefore hamper the remaining CH attacks that would not be prevented by regular stimulation (Fig. 1). This hypothesis is supported by our current subjects mentioning that voltage tuning prevents and ceases additional CH attacks. This phenomenon can likely be linked to the presence of a so called ‘threshold’, which is dynamic and must be exceeded by electrical signals, in order to successfully prevent a CH attack from occurring (Fig. 1).

Previous data revealing a correlation between severity of CH attack and neuronal activity, measured by cerebral blood flow, suggest that this threshold may depend on the intensity of the CH attack [27]. Furthermore, other studies demonstrate enhanced release of certain neuropeptides, such as calcitonin gene-related peptide (CGRP) and vasoactive intestinal peptide (VIP), during severe CH attacks [28–32]. This suggests a potential increase in the threshold for effective electrical stimulation with the rising intensity of CH attacks. Additionally, studies showing decreased neuropeptide release like substance P in the TCC after administration of sumatriptan or oxygen, also support for this mechanism [30, 33, 34].

Together, these findings bolster the hypothesis that neuropeptide levels may play a role in CH attack severity. Furthermore, previously published data show that electrical stimulation with higher amplitudes may be related to greater pain reductions as compared to stimulation with lower amplitudes [35]. Based on these findings, it may be suggested that in case of severe CH attacks, a higher threshold must be exceeded in order to prevent these attacks from occuing. This would imply that the benefit of voltage tuning is caused by its effect on severe CH attacks with a threshold that can otherwise not be exceeded by regular stimulation settings. Furthermore, it might also suggest that, in order for voltage tuning to be effective, a minimal increase in amplitude must be achieved and maintained for a substantial amount of time. This is proposed by one of the patients reporting that voltage tuning for a shorter duration, causes a recurrence of CH attacks (Table 1). Current outcomes show variability in duration, frequency, and amplitude increase during voltage tuning, which may be attributed to the lack of a standardized approach to tuning, variability in CH attacks and heterogeneity between patients.

As the mechanism of voltage tuning is likely multifactorial, additional behavioral factors may also be involved. Studies showing that CH attacks are associated with enhanced stress and depression [36, 37], may support the involvement of emotion and cognition in voltage tuning. The implication of emotion is supported by patients in the current sample describing a decrease in anxiety during moments of voltage tuning. The effects of voltage tuning may also be due to enhanced sense of self-control of CH attacks. Furthermore, voltage tuning provokes a sensation of numbness, therefore making patients more aware that their stimulator is on, consequently leading to patients feeling more confident with their implant. Taken together, it seems that the efficacy of ONS and potential of voltage tuning is possibly determined by a complex interplay between cognition and emotion. From a neurophysiological point of view, the balance between threshold and amplitude in stimulation are also considered key components to this behavior.

Promising effects of voltage tuning raise questions as to whether constant stimulation at higher voltages would lead to a further decrease in CH attacks. Based on the theory of the threshold in voltage tuning, it can be suggested that high amplitudes will provide further reductions in the short-term. Although some papers have suggested the presence of habituation in patients undergoing neuromodulation [38–40], we hypothesize that voltage tuning, which includes adjusting the amplitude solely during CH attacks, is more effective as compared to constant high amplitudes, as it is assumed to prevent the occurrence of habituation. Moreover, patients in the current study also declared that once the attack has disappeared, high amplitudes cause an overstimulation in the occipital region which is considered as unwanted and can solely be tolerated for a short-duration, hence further supporting the preference of voltage tuning over constant stimulation with higher amplitudes.

Another important factor to be considered in voltage tuning, is the IPG. Increasing the amplitude on a regular basis likely requires more frequent charging of rechargeable IPG’s and earlier replacement of the non-rechargeable variants, which may be considered a burden for patients [41, 42]. Nevertheless, current outcomes raise further interest in the use of ONS as acute attack treatment in patients with CH. Voltage tuning may be an alternative approach to other acute attack treatments, like oxygen or sumatriptan, and is therefore useful in reducing pharmacological side effects such as injection site reactions, drowsiness, and feeling of weakness [12, 13]. Future studies are required to compare the efficacy of voltage tuning with standard pharmacological acute attack treatments. To date, this is the first study to describe individuals with CH managed with ONS who adjust their amplitude to manage their headaches. As voltage tuning appears to be beneficial for a subset of patients with CH, this behavior should be further investigated.

Various limitations like heterogeneity in CH subtypes and wide range of follow-up were present, which can likely be attributed to baseline data being collected retrospectively.

With regard to the wide range in follow-up (6–42 months), this might have affected the clinical outcomes due to possible alterations in efficacy of ONS long term [40]. We were aware of this prior to analysis, however, as this was exploratory and a descriptive study with the assessment of voltage tuning being primary aim and the sample size otherwise being small, we chose to include various follow-up moments. Due to the sample size, corrections for confounders like age, gender and BMI, could not be made. Hence, conclusions should be taken with caution. Moreover, the reason for lack of effect of voltage tuning in a subset of patients could not be assessed due to limited data availability in intensity, frequency, and duration of voltage tuning.

A major strength of this study is that all subjects were implanted using the same standardized surgical approach and team. Hence, effects of variable electrode placement, imaging and patient positioning on outcomes did not affect the current analysis. Another aspect providing strength to this study, includes the subdivisions of efficacy of ONS into four groups, as it allows for the assessment of voltage tuning in both responders and non-responders of ONS. Further, though medication use was measured in the current study, we did not correct for the intake when analyzing the effects of voltage tuning. Using a clear response definition for the analysis was considered a major strength of this study. However, important to note is that our response rate only includes patients having ≥ 50% reduction in attack frequency, intensity and duration. However, those with < 50% improvement are also likely to benefit from ONS, especially when considering the severity of the CH attacks. As a result, even a few reductions in CH attacks may result in significant improvements in the quality of life, even in patients defined as non-responders. Though, using 50% as a cut off value diminishes inclusion of placebo effect, it might have led to an underestimation of the overall efficacy of ONS as most other studies use a 30% cut-off value instead [2, 43]. Moreover, ONS has also shown to have beneficial effects on overall quality of life, however, as this was not assessed in the current study, the effect of ONS and voltage tuning might be underestimated [15]. The occurrence of voltage tuning might also been affected by some patients being unaware of the possibility of voltage tuning up until te interview. The current analysis shows that some patients suffering unilateral attacks, performed tuning solely on the attack site, whereas others adjusted the amplitude on both sites. Though an interesting finding, it was not further investigated in the current study, however forms an interesting field for future research as it may improve our understanding of the mechanism and optimal approach to voltage tuning.

### Future directions

To further assess the current phenomenon of voltage tuning, prospective studies evaluating QoL, efficacy as well as site of voltage tuning, using control groups are necessary.

## Conclusion

ONS is an effective approach for therapy resistant CH due to its effect on attack frequency, intensity and duration. Despite successful outcomes of ONS, some patients still experience CH attacks at times, which may be managed by voltage tuning instead of oxygen therapy or sumatriptan injection or nasal spray. Outcomes show that voltage tuning may cease and/or terminate CH attacks and therefore raise interests in the use of ONS as acute attack treatment for patients with resistant CH treated with ONS. While the working mechanism of voltage tuning remains unknown, it is possible that some sort of neurophysiological ‘threshold’ has to be reached to prevent a new attack. The heterogeneity in ‘stimulation behavior’ that is observed in ONS treatment probably follows the complex interplay between cognition and emotions in patients with CH. Future research should be performed to further investigate the optimal settings as well as occurrence and potential of voltage tuning in the treatment of refractory CH.

## Data Availability

No datasets were generated or analysed during the current study.

## Abbreviations

CH: Cluster headache
DNIC: Diffuse noxious inhibitory control
IPG: Implantable pulse generator
NRS: Numeric rating scale
ONS: Occipital nerve stimulation
SCS: Spinal cord stimulation
TCC: Trigeminal cervical complex
VNS: Vagus nerve stimulation
